# Effects of Er-Miao-San extracts on TNF-alpha-induced *MMP-1* expression in human dermal fibroblasts

**DOI:** 10.1186/0717-6287-48-8

**Published:** 2015-01-26

**Authors:** Seunghee Bae, Younjung Jung, Yeong Min Choi, Shunhua Li

**Affiliations:** Korea Institute for Skin and Clinical Sciences and Molecular-Targeted Drug Research Center, Konkuk University, Seoul, Republic of Korea; Department of Bioengineering, Graduate School of Konkuk University, Seoul, Republic of Korea; Department of Beauty Education, Graduate School of Education, Konkuk University, 120 Neungdong-ro, Gwangjin-gu, Seoul, 143-701 Republic of Korea

**Keywords:** Er-Miao-San, *MMP1*, Human dermal fibroblasts, TNF-α, NF-κB

## Abstract

**Background:**

Various health benefits have been attributed to Er-Miao-San (EMS), a traditional Chinese herbal formulation that contains equal amounts of cortex phellodendri (*Phellodendron amurense* Ruprecht) and rhizoma atractylodis (*Atractylodes lancea* D.C). However, its effect on the anti-inflammatory activity in human dermal fibroblasts (HDFs) and the mechanism underlying this effect are unknown.

**Results:**

This study investigated the effects of EMS on TNF-α-induced *MMP-1* expression in HDFs. Our data show that EMS inhibited TNF-α-induced *MMP-1* expression in a concentration-dependent manner. Interestingly, EMS maintained IκB content without inhibiting the phosphorylation of MAPKs, which are well-established upstream kinases of NF-κB. Moreover, EMS reduced the level of nuclear p65 protein in HDFs. Luciferase assay revealed that EMS inhibits the transcriptional activity of NF-κB by stabilizing IκB. Our results show that EMS exerts its anti-inflammatory effect by inhibiting NF-κB-regulated genes such as *IL-1β* and *IL-8*. Moreover, EMS effectively inhibited TNF-α-induced expression of *MMP-1* via the NF-κB pathway.

**Conclusions:**

Taken together, our data suggest that EMS could potentially be used as an anti-inflammatory and anti-aging treatment.

## Background

Skin aging can be divided into two types, namely intrinsic aging, which is caused by the natural consequences of physical change, and extrinsic aging, which is caused by exposure to environmental factors such as ultraviolet (UV) rays and pollutions [1]. Oxidative stress due to UVB, an extrinsic aging factor, causes DNA damage, and leads to human dermal fibroblast (HDF) senescence [2]. In addition, inflammation of skin cells caused by various environmental factors is also known to be a representative factor that induces skin aging [3].

Wrinkle formation is the most representative characteristic of skin aging, and is closely related to a reduction in skin elasticity and degeneration of the extracellular matrix (ECM) [4]. The ECM consists of a mesh of fibrous proteins, such as collagen, elastic fibers, and glycosaminoglycans, which are generated by fibroblasts. These cells synthesize procollagen type-1 (Col-1) and type-3 (Col-3) in the dermis and secrete matrix metalloproteinase (MMP), an enzyme that degrades nearly all ECM components, including collagen. Wrinkle formation is caused by the secretion of higher levels of MMP-1 and reduction of procollagen synthesis in HDFs [5]. Several compounds including cordycepin and brazilin have recently been suggested as possible anti-aging agents through suppressing the secretion of MMP-1 and MMP-3 in HDFs [6, 7].

NF-κB is one of transcription factors and forms homo-and heterodimer complexes with Rel family proteins such as RelA (p65), RelB, cRel, p50, and p52 [8]. The transcriptional activity of NF-κB is mainly regulated by its intracellular localization, which is primarily controlled by inhibitor of κB (IκB) [8]. IκB can dimer with NF-κB, which induces cytoplasmic retention of NF-κB [9]. Upon lipopolysaccharide (LPS) or cytokine stimulation, IκB kinase (IKK) is activated and phosphorylates IκB, and then phosphorylated IκB underwent polyubiquitination-mediated proteasomal degradation [10]. Following the degradation of IκB, NF-κB translocates to the nucleus and induces transcription of various inflammatory genes, including *interleukin (IL)-1β* and IL-8 [11, 12]. Accumulating studies have also shown that NF-κB regulates skin aging by regulating the expression level of *MMP-1* in dermal fibroblasts [13, 14]. Interestingly, it was reported that suppression of NF-κB activation reduces *MMP-1* expression in HDFs and inhibits skin photoaging [15]. Furthermore, inflammation-induced activation of NF-κB causes deterioration of dermal tissue by promoting the expression of *MMP-1*, which exerts degradation of dermal type I collagen [16].

Er-Miao-San (EMS) is a compound commonly found in traditional Chinese medicine (TCM) that consists of equal amounts of *Cortex Phellodendri* (CP) and *Rhizoma Atractylodis* (RA). The major component of CP, berberine, promotes the apoptosis of cancer cells by regulating caspase-3 [17]. In 3 T3-L1 adipose cells, free fatty acid-induced insulin resistance was recovered by berberine through activation of inhibitor of κB kinase-β (IKK-β) [18]. Moreover, berberine prevents receptor activator of nuclear factor kappa-B ligand (RANKL)-induced NF-κB activation by blocking phosphorylation of inhibitor of κBα (IκBα) [19]. RA extract has been known to inhibit the activity of cyclooxygenase-1 (COX-1) [20], 15-lipoxygenase [21], and thromboxane [22], as well as block the expression of interleukin (IL)-1β/IL-6 [23] and IL-2 [24]. Studies have shown that RA also inhibits NF-κB [25], and that EMS exerts beneficial effects on prevention of cancer progression, inflammation, atherosclerosis, and arthritis [26, 27]. However, little is known about the biological effects of EMS on skin aging.

TNF-alpha (TNF-α) is one of the major inflammatory cytokines [28]. It was reported that TNF-α induces *MMP1* expression and suppresses collagen synthesis in HDFs [28]. After TNF-α stimulation in cells, NF-κB is activated and acted as a transcription factor for *MMP1* expression [8, 13]. Besides IκB, mitogen-activated protein kinases (MAPKs) are important signaling molecules that affect NF-κB activation [29], as evidenced by the lack of NF-κB transactivation following MAPK inhibition [29]. Here, we demonstrated that treatment with EMS inhibits TNF-α-induced *MMP-1* expression through suppressing NF-κB nuclear localization in HDFs. Also, we observed that EMS-mediated NF-κB inhibition was not dependent on MAPK signaling pathways in HDFs.

## Results

### Effect of EMS on cell viability

**Figure 1:**
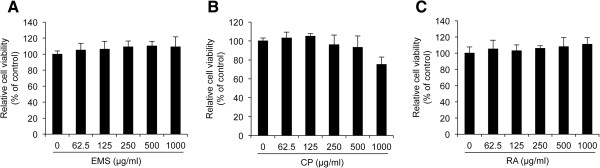
**The effect of EMS on cell viability in HDFs.** Cell viability was determined by the WST-1 assay. HDFs were incubated with 62.5-1000 μg/ml EMS **(A)**, Cortex Phellodendri (CP) **(B)**, or Rhizoma Atractylodis (RA) **(C)** for 24 h. The graph represents mean ± S.D. of relative cell viability from three independent experiments.

We first investigated whether the treatments of EMS, CP and RA on human skin dermal fibroblasts will induce cytotoxic or non-cytotoxic properties, and what are the non-cytotoxic concentration ranges of EMS, CP and RA on human skin dermal fibroblasts before further experiments that investigate the EMS-induced protection effect on TNF-α-induced *MMP-1* expression. Therefore, we set the experimental conditions as followed; the treatment concentrations is 0 – 1000 μg/ml, and the treatment time is 24 h. To examine the cytotoxicity of EMS, CP, and RA, HDFs were treated with various concentrations (0–1000 μg/ml) of the reagents for 24 h, and the WST-1 assay was performed to evaluate cell viability. As shown in Figure 1A and C, treatment with EMS and RA slightly increased the cell viability of HDFs as the concentration increased. Interestingly, treatment with less than 500 μg/ml CP did not induce any significant cytotoxicity relative to the control; however, the viability was decreased to 75.1% when HDF cells were treated with 1000 μg/ml CP, respectively (Figure 1B). Therefore, we concluded that although larger dose of CP (1000 μg/ml) show cytotoxicity in HDFs, EMS, which contain equal amount of CP and RA, is not cytotoxic reagent in HDFs, and the doses of 250 and 500 μg/ml EMS, CP and RA were used in further experiments.

### Effect of EMS on TNF-α-induced expression of *MMP-1*mRNA

**Figure 2:**
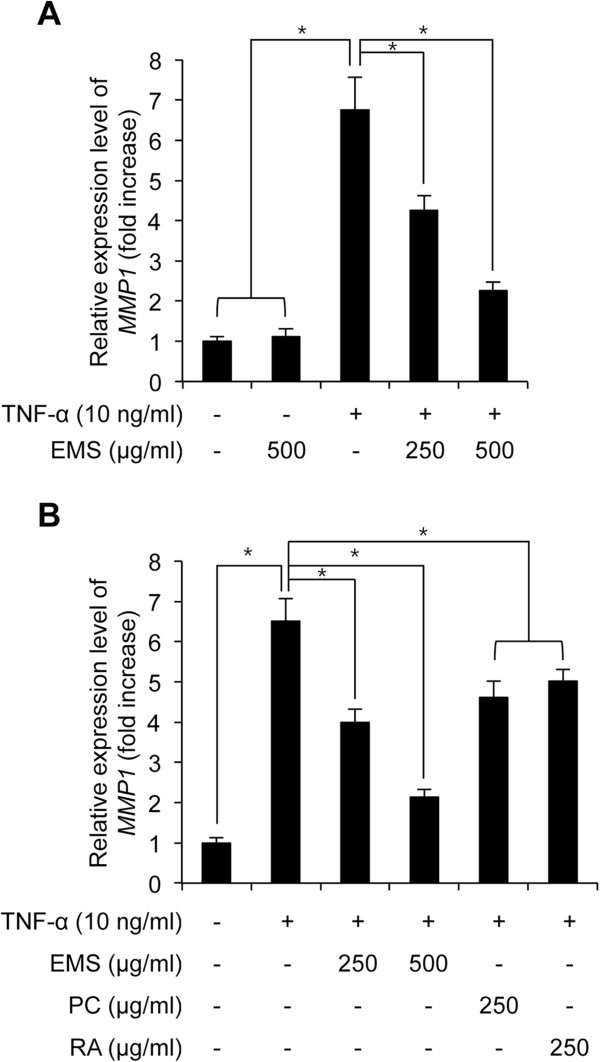
**The effect of EMS on TNF-α-induced** ***MMP-1*** **mRNA expression in HDFs.** HDFs were pre-treated with 250–500 μg/ml EMS **(A)**, 250 μg/ml CP and 250 μg/ml RA for 3 h **(B)**, and subsequently stimulated with 10 ng/ml TNF-α for 4 h. The level of *MMP-1* mRNA was determined by qRT-PCR. Independent experiments were performed in triplicate (*p < 0.05).

We next investigated whether TNF-α-induced downregulation of *MMP1* expression could be regulated by treating EMS in HDFs. HDFs were seeded and pretreated with 250 and 500 μg/ml EMS for 3 h, and then exposed to TNF-α for 4 h. After TNF-α stimulation, cells were gathered and the expression level of *MMP1* was investigated using RT-PCR with its specific primers. As shown in Figure 2A, TNF-α increased the expression level of *MMP1* by 6.75 ± 0.81 fold compared with non-treated control cells. However, 250 and 500 μg/ml EMS treatments before TNF-α stimulation significantly inhibited the expression level of *MMP1* by 4.26 ± 0.37 and 2.26 ± 0.21 fold, respectively, as compared with non-treated control cells (Figure 2A). Of note, the upregulated MMP1 expression induced by TNF-α was largely downregulated to 33.57% in 1000 μg/ml EMS pretreated HDFs as compared with TNF-α treated cells. Furthermore, we investigated which component between CP and RA could regulate the TNF-α-induced MMP1 expression. HDFs were seeded and pretreated with 250 μg/ml CP and RA for 3 h, and then exposed to TNF-α for 4 h. As shown in Figure 2B, both CP and RA pretreatments significantly inhibited the TNF-α-induced *MMP1* upregulation in HDFs. Overall, we conclude that EMS pretreatment significantly inhibits TNF-α-induced *MMP1* expression in HDFs.

### Effect of EMS on IκB degradation and NF-κB transactivation activity

**Figure 3:**
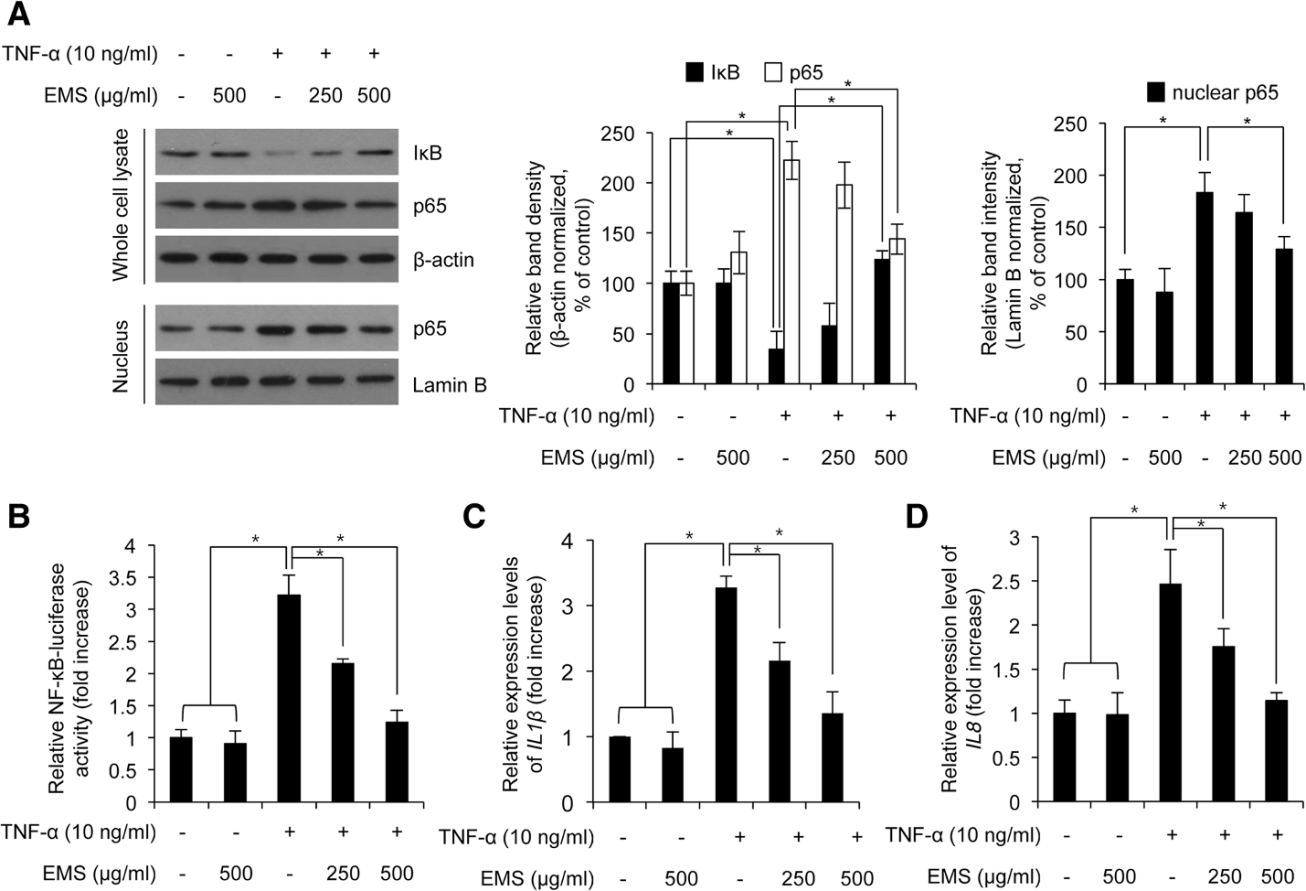
**The effect of EMS on IκB degradation and NF-κB transactivation in TNF-α-induced HDFs.** HDFs were pre-treated with 250–500 μg/mL EMS for 3 h, and subsequently stimulated with 10 ng/mL TNF-α for 4 h. **(A)** IκB and p65 protein levels were analyzed by western blot. Nuclear p65 protein was analyzed using nuclear extracts. Lamin B was used as the loading control. The relative expression ratio of each band was calculated after densitometric analysis using image J software and normalization to β-actin or Lamin B signal. *p < 0.05 compared with control or TNF-α-treated group. **(B)** NF-κB transactivation activity was determined by luciferase assay using NF-κB reporter NIH-3 T3 cells. **(C)** The level of *IL-1β* mRNA was determined by real-time qPCR. **(D)** The level of *IL-8* mRNA expression was determined by real-time qPCR. Independent experiments were performed in triplicate (*p < 0.05).

We investigated whether EMS-mediated inhibition of TNF-α-induced MMP1 expression is associated with NF-κB activity in HDFs. First, we confirmed that TNF-α stimulation decreased the protein level of IκB in HDFs (Figure 3A, lane 1 and 3). Next, to investigate the effect of EMS on TNF-α-induced IκB degradation, HDFs were seeded and pretreated with 250 and 500 μg/ml EMS before TNF-α stimulation. As shown Figure 3A, pretreatment with EMS rescued the decreased IκB protein mediated by TNF-α stimulation in HDFs. Third, we also found that the total and nuclear protein level of p65, which is subunit of NF-κB [8], was increased by TNF-α stimulation and the increased p65 protein was largely decreased by EMS pretreated HDFs, indicating that TNF-α-induced NF-κB nuclear translocation was inhibited by EMS treatment in HDFs (Figure 3A). Lastly, we further examined whether EMS-mediated inhibition of TNF-α-induced p65 nuclear translocation is indeed related with transcriptional activity of NF-κB in HDFs. Using NF-κB luciferase reporter NIH-3 T3 fibroblasts, we investigated the NF-κB activity after EMS and TNF-α treatment. As shown in Figure 3B, pretreatment with EMS significantly decreased the upregulated luciferase activity mediated by TNF-α, indicating that EMS inhibits TNF-α-induced NF-κB activation. Notably, TNF-α only stimulation increased the luciferase activity to 3.21 ± 0.31 compared with non-treated control cells; however, EMS pretreatment before TNF-α stimulation increased the luciferase activity only to 2.16 ± 0.07 and 1.23 ± 0.19 fold compared with non-treated control, respectively (Figure 3B). Therefore, we conclude that EMS inhibits TNF-α-induced NF-κB activation and MMP1 expression in HDFs.

NF-κB activates transcription of *IL-1β* and *IL-8* by binding to their promoters [11, 12], leading to inflammation and degradation of the tissue matrix structure by inducing the formation of inflammatory mediators such as COX-2, PGE2, and MMP [30]. Thus, we performed qRT-PCR to examine the effect of EMS on IL-1β and IL-8 expression. Our results indicate that the level of IL-1β and IL-8 mRNA increased following TNF-α treatment, but was decreased by EMS in a dose-dependent manner (Figure 3C and D).

### Effects of EMS on phosphorylation of MAPK pathway

**Figure 4:**
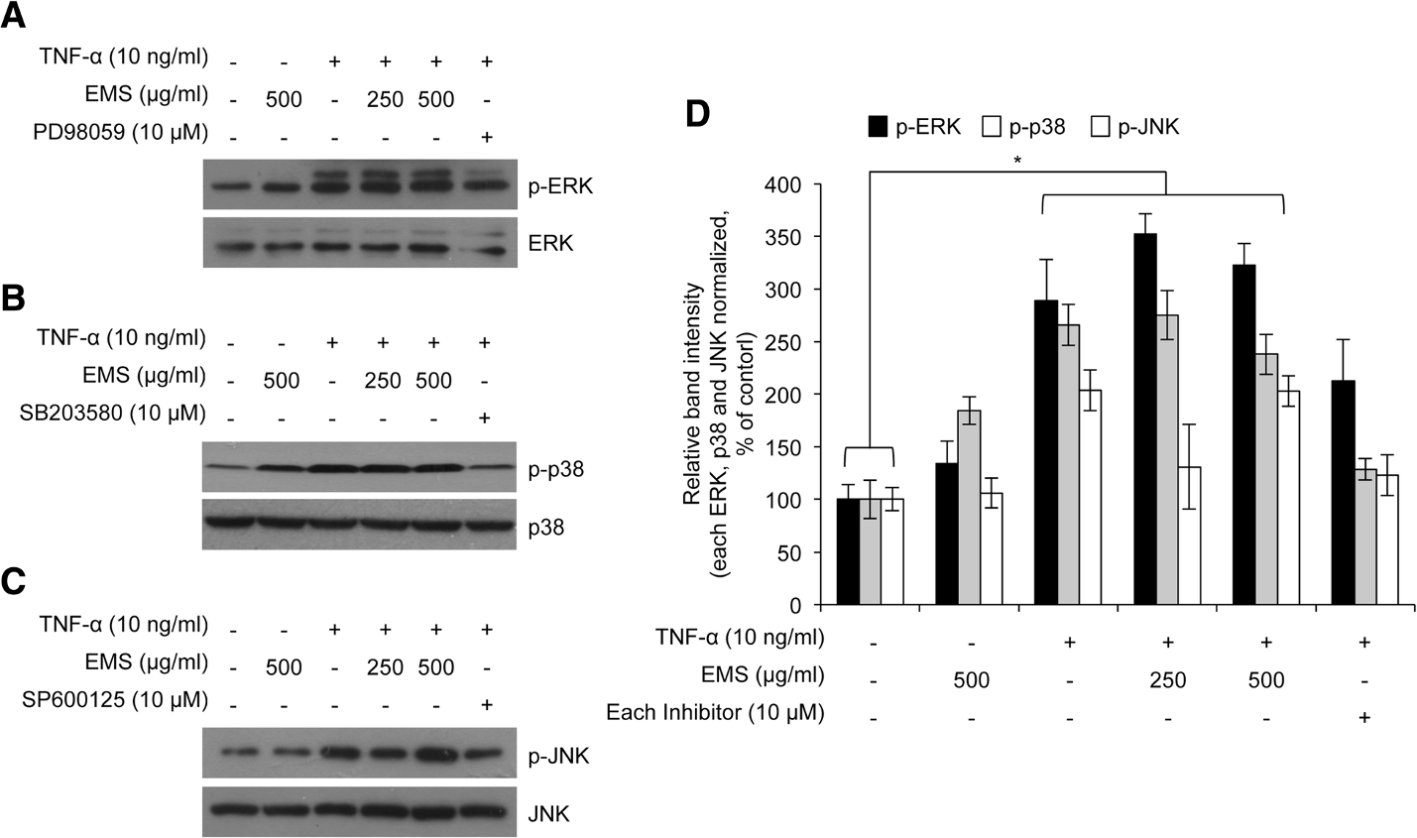
**The effect of EMS on phosphorylation of MAPK in TNF-α-induced HDFs.** The phosphorylation of ERK, p38, and JNK was analyzed by western blot analysis. As controls, cells were treated with 10 μM PD98059 (an ERK inhibitor) **(A)**, SB203580 (a p38 inhibitor) **(B)**, or SP600125 (a JNK inhibitor) **(C)**. **(D)** The relative expression ratio of each p-ERK, p-p38 and p-JNK was calculated after densitometric analysis using image J software and normalization to each ERK, p38 and JNK signal. *p < 0.05 compared with non-treated control group.

To investigate the effect of EMS on the MAPK pathway, ERK, JNK, and p-38 phosphorylation were investigated after treatment with EMS and TNF-α in HDFs. Immunoblotting results showed that pretreatment with EMS did not altered the TNF-α-induced phosphorylation of ERK, JNK, and p-38 in HDFs (Figure 4). Therefore, these results showed that the intracellular mechanism of EMS-mediated inhibition of TNF-α-induced NF-κB activation and *MMP-1* expression is independent of the MAPK signaling cascade in HDFs.

## Discussion

Skin is constantly in direct contact with the environment, thereby inducing aging and skin damage [1]. Wrinkle formation, the most representative characteristic of skin aging, is caused by oxidative stress and inflammation of skin cells. Thus, compounds that exhibit an anti-inflammatory effect on skin cells have been suggested as promising anti-aging agents [6, 7]. In this study, we investigated the inhibitory effect of EMS on inflammation-induced skin aging. Therefore, examination of the effect of EMS on TNF-α-induced *MMP-1* expression in HDFs indicated that pre-treatment with EMS for 3 h decreased *MMP-1* mRNA in a concentration-dependent manner (Figure 2A). CP and RA treatment also decreased the expression level of *MMP-1* mRNA (Figure 2B), suggesting that these active ingredients might synergistically inhibit TNF-α-induced *MMP-1* expression.

The expression of *MMP-1* in response to inflammation is regulated by the transcription factor NF-κB. In HDFs, NF-κB activation is involved in regulating inflammation through various intracellular signaling pathways, including the MAPK pathway [30]. MAPKs constitute a group of serine/threonine protein kinases that can be subdivided into three subfamilies: p42/p44 ERK, JNK, and p38 MAPK. MAPKs are activated by various extracellular stimuli and induce the phosphorylation of key signaling molecules associated with cell proliferation, inflammation, and apoptosis [31]. As shown in Figure 4A, B and C, we found that EMS inhibited TNF-α-induced activation of NF-κB. Also, we found that EMS inhibited TNF-α-induced *MMP-1* expression. The phosphorylation of MAPKs, which is one of NF-κB activators, was also known to be regulated by TNF-α stimulation [29]. However, we found that the increased levels of phosphorylation of MAPKs by TNF-α stimulation were not changed in EMS-treated cells. Those results indicated that EMS-dependent *MMP-1* expression is not related with the phosphorylation of MAPKs, and EMS-dependent NF-κB activation would not be dependent on the phosphorylation level of MAPKs. Therefore our results suggest that EMS inhibits the NF-κB pathway independent of the MAPK pathway.

Association of IκB with the NF-κB p65/p50 dimer plays an important role in regulating the nuclear translocation and target gene transcription by NF-κB. It is well established that IκB degradation induces the nuclear translocation of p65 [8]. Thus, we assessed the level of IκB in TNF-α-stimulated HDFs following EMS treatment. Our data show that EMS treatment increased the level of IκB and decreased nuclear p65 (Figure 3A). Nuclear translocation of NF-κB due to IκB degradation is essential for activating NF-κB [8]. We further explored this situation by assessing NF-κB transactivation activity. NF-κB transactivation was found to be increased by TNF-α treatment, but significantly decreased by EMS in a concentration-dependent manner (Figure 4B). In addition, EMS inhibited the expression of *IL-1β* and *IL-8*, which are regulated by NF-κB (Figure 4C,D). In conclusion, our results demonstrate that EMS exerts its anti-inflammatory effect on skin by inhibiting the TNF-α-induced expression of *MMP-1* in HDFs by blocking NF-κB. EMS reduces inflammation by increasing the level of IκB present in the cell, which reduces p65 nuclear translocation and thus NF-κB transactivation. Also, several reports have showed that inhibition of NF-κB activation suppresses *MMP-1* expression in several cells including human dermal fibroblasts [32, 33]. Our data showed that EMS-dependent loss of *MMP-1* expression was mediated by EMS-mediated inhibitory effect on NF-κB activation. Therefore, our results also suggest that the effect of EMS-dependent loss of MMP1 expression might be strengthened by treatment with NF-κB inhibitors.

## Conclusions

In summary, we examined the inhibitory effect of EMS on inflammation-induced NF-κB activation and MMP1 generation in HDFs. Our data suggest that EMS is a potential anti-aging agent against inflammation-induced skin aging.

## Methods

### Herbal extraction and characterization

Dried CP and RA were obtained from the oriental pharmacy in Kyung Hee Hospital of Oriental Medicine, Kyung Hee University (Seoul, Korea). The each dried sample (20 g) was added to 200 ml sterilized distilled water (DW) to produce a 10% solution. For EMS, equal amounts (10 g) of the dried CP and RA were added to 200 ml sterilized DW to be 10% solution. Those solutions were subsequently extracted for 24 h at 60°C and then filtered. After the extraction and filtration, the extracts were subjected to vacuum evaporating and freeze-drying. Finally, we obtained 3.043 g of EMS, 2.548 g of CP, and 2.997 g of RA, respectively. These powders were dissolved into the tissue cell culture medium and used for experiments.

### Cell culture and treatment

HDFs were obtained from Lonza (Basel, Switzerland) and cultured in Dulbecco's Modified Eagle Medium (DMEM; WELGENE Inc., Daegu, Korea) supplemented with 10% fetal bovine serum, 100 U/ml of penicillin, and 100 μg/ml of streptomycin. The cells were maintained at 37°C in a 5% CO_2_ incubator. TNF-α was purchased from Sigma-Aldrich (St. Louis, Mo, USA). Cells (1 × 10^6^ or 3 × 10^5^) were seeded in 60 pi or 6-well cell culture dishes and incubated for overnight. After incubation, the cells were treated with 10 ng/ml TNF-α for 4 h in a serum-free media. All experiments handling human cells were carried out in line with the Tenets of the Declaration of Helsinki.

### Quantitative real-time PCR analysis

cDNAs were synthesized from total RNA using M-MLV reverse transcriptase (Enzynomics, Seoul, Korea) according to the manufacturer’s protocol. The forward and reverse primers for human *MMP-1* were 5′-TCTGACGTTGATCCCAGAGAGCAG-3′ and 5′-CAGGGTGACACCAGTGACTGCAC-3′, respectively. The forward and reverse primers for human *β-actin* were 5′-GGATTCCTATGTGGGCGACGA-3′ and 5′-CGCTCGGTGAGGATCTTCATG-3′, respectively. The forward and reverse primers for human *IL-1β* were 5′-ACAGATGAAGTGCTCCTTCCA-3′ and 5′-GTCGGAGATTCGTAGCTGGAT-3′, respectively. Also the forward and reverse primers for human IL-8 were 5′-ATGACTTCCAAGCTGGCCGTGGCT-3′ and 5′- TCTCAGCCCTCTTCAAAAACTTCTC-3′, respectively. PCR was performed using the HOT FIREPol EvaGreen PCR Mix Plus (Solis BioDyne, Estonia) with Line gene K software (Bioer Technology Co., Ltd., Hangzhou, China). The C_T_-value for *MMP-1*, *IL-1β* and *IL-8* were normalized to *β-actin*. The 2^-ΔΔCt^ method was used to calculate relative expression level of *MMP-1, IL-1β* and *IL-8*. Data were presented as mean ± S.D. (n = 9; three independent experiments).

### Cell viability assay

Cell viability was assessed using the WST-1 assay according to the manufacturer’s instructions (Itsbio, Seoul, Korea). The results are represented graphically as the measured cell viability ratio normalized to the control.

### Western blotting

Cellular proteins was extracted by lysis buffer (50 mM Tris–HCl, pH 8.0, 150 mM NaCl, 1% NP-40, 0.1% SDS, and 0.5% sodium deoxycholate) and EDTA-free protease and phosphatase inhibitor cocktail (Roche, Switzerland). Equal amounts of protein samples were separated by 10% sodium dodecyl sulfate (SDS) polyacrylamide gel electrophoresis and then transferred onto nitrocellulose membrane (Whatman Protan BA83, GE Healthcare Life Science, Freiburg, Germany). After blocking with 5% skim milk for 1 h at room temperature, the membranes were incubated first with primary antibody at 4°C overnight and subsequently with peroxidase-conjugated secondary antibody at room temperature for 1 h. The protein bands were detected using enhanced chemiluminescence reagents. Primary antibody specific for MMP1 NF-κB-p65, IκB and Lamin B were purchased from Santa Cruz Biotechnology (Santa Cruz, CA, USA). Anti-β-actin antibody was purchased from Sigma-Aldrich (St. Louis, MO, USA). Anti-p38, p38, pERK, ERK, pJNK and JNK were purchased from Cell Signaling Technology (Danvers, MA, USA).

### Preparation of nuclear protein extracts

Cells were gently resuspended in 500 μl of buffer A (10 mM HEPES, pH 7.5, 1.5 mM MgCl_2_, 10 mM KCl, 0.5 mM DTT, and 0.05% NP-40) and then incubated for 20 min on ice. The cells were centrifuged for 5 min at 3,000 × g at 4°C. Then, 25 μl of buffer B (1% Triton X-100, 300 mM NaCl, 5 mM HEPES, pH 7.5, 1.5 mM MgCl_2_, 0.2 mM EDTA, 0.5 mM DTT, and 26% glycerol) was added, and the samples were mixed prior to centrifugation for 20 min at 12,000 × g at 4°C. The supernatant (nuclear protein extract) was collected and stored at -80°C until use.

### NF-κB transactivation activity assay

NF-κB-luciferase reporter stable NIH-3 T3 cell line, which was stably transfected with the NF-κB-luciferase reporter vector (pNF-κB-luc; Affymatrix-Panomics, Santa Clara, CA, USA), was purchased from Affymatrix-Panomics. pNF-κB-luc was designed to measure the transcriptional activity of NF-κB. Six copies of NF-κB binding sequences (5′-GGGAATTTCCGGGAATTTCCGGGAATTTCCGGGAATTTCCGGGAATTTCCGGGAATTTCC-3′) were subcloned into the upstream region of luciferase cDNA. The NIH-3 T3/NF-κB-luc cell line was obtained by co-transfection of pNF-κB-luc (Affymatrix-Panomics) and pHyg into NIH-3 T3 cells, followed by hygromycin selection. To test NF-κB activity, approximately 1 × 10^5^ NF-κB reporter NIH-3 T3 stable cells were seeded onto 60-mm culture dishes and cultured for 24 h. Cells were then lysed by adding Passive Lysis Buffer (Promega, Madison, WI, USA) and incubating for 30 min on ice. After centrifugation for 30 min at 12,000 × g at 4°C, the supernatant was collected. The cell lysate was treated with luciferin (Promega) and its luminescence was measured using a Veritas Luminometer (Turner Designs, Sunnyvale, CA, USA). Results shown are the averages of three independent experiments.

### Statistical analysis

A result of three observations per group was subjected to a statistical analysis. Data are presented as mean ± the standard deviation (S.D.). Statistical analysis was performed using two-tailed Student’s *t*-test analysis. P < 0.05 was considered significant.
